# Transforming Hemophilia A Care: Insights into New Therapeutic Options

**DOI:** 10.3390/life14121568

**Published:** 2024-11-29

**Authors:** Iasmina-Maria Iurea, Emilia Severin, Alexandra Matei

**Affiliations:** Genetics Department, Carol Davila University of Medicine and Pharmacy, Dionisie Lupu 37 Street, 020021 Bucharest, Romania; iasmina-maria.iurea0720@stud.umfcd.ro (I.-M.I.); alexandra.matei0720@stud.umfcd.ro (A.M.)

**Keywords:** hemophilia A, bleeding disorder, hemophilia genetics, gene therapy, quality of life

## Abstract

Hemophilia A is a hereditary bleeding disorder characterized by a deficiency in clotting factor VIII, leading to significant morbidity and a reduced quality of life. This review provides an updated overview of the current understanding of hemophilia A, highlighting its genetic underpinnings and advancements in treatment strategies. A literature review was conducted using various available databases. Relevant studies on hemophilia A, covering genetics and treatment options, were selected and summarized. Recent developments in gene therapy are discussed, showcasing their potential to offer long-term solutions and reduce the burden of treatment. Additionally, the review addresses global disparities in care and policy implications, emphasizing the need for comprehensive healthcare frameworks to improve outcomes for individuals living with hemophilia A worldwide. By synthesizing recent findings and insights, this review aims to inform clinicians and policymakers about the evolving landscape of hemophilia A management and the necessity for equitable access to care.

## 1. Introduction

Hemophilia A, or classic hemophilia, is a hereditary bleeding disorder characterized by spontaneous or prolonged hemorrhages due to a deficiency of coagulation factor VIII. Factor VIII, a large plasma glycoprotein, is encoded by the F8 gene located on the X chromosome (Xq28). The estimated prevalence is approximately 1 in 6000 live male births, without significant racial or ethnic variation [1]. Very rarely, females can be affected by hemophilia A. Typically, females have two X chromosomes, and one of them undergoes random inactivation in each cell (X chromosome inactivation). If the X chromosome carrying the healthy F8 gene is predominantly inactivated, the defective X chromosome becomes functionally dominant, leading to a significant reduction in factor VIII levels and causing symptoms of hemophilia A in females.

Hemophilia A falls under a broader classification of hemophilia, which includes hemophilia B, characterized by a deficiency in factor IX. Hemophilia A is classified as severe, moderate, or mild depending on the level of the missing clotting factor (the lower the level, the more bleeding problems the affected person will have without treatment) [1,2]. Hemophilia A is the most common form, accounting for about 80% of cases. Factor VIII does not cross the placenta; as a result, the condition can be detected at birth based on the absence or low levels of coagulant factor activity. Prenatal diagnosis is possible if there is a family medical history of the condition.

Hemophilia A is classified under ICD-11 code 3B10.0, OMIM entry 306700, and ORPHA code 98878.

The historical context of hemophilia A is intriguing, with references to bleeding disorders traceable back to ancient times. In the second century AD, Jewish scholars would exempt a male infant from circumcision if his mother and her sisters had lost other sons due to bleeding after circumcision [3,4]. This practice indirectly recognized the X-linked inheritance pattern. However, it was not until the 19th century that the condition was formally identified, and the discovery of factor VIII in 1937 marked a pivotal moment in understanding this disorder. In the 1950s, the X-linked inheritance pattern was established, explaining why hemophilia predominantly affects males, while females often serve as carriers.

In Europe, cases of hemophilia have been described among several branches of royal families in England, Germany, Russia, and Spain. However, it was unclear whether these cases represented hemophilia A or B [5] until the 21st century [6,7]. One such case involved Alexis Romanov, the son of Tsar Nicholas II, who was stricken by the disease. His condition revealed a pathogenic variant in the splice acceptor site of exon 4 in the F9 gene, responsible for hemophilia B. This variant had a significant impact on European royal families throughout the 19th and 20th centuries [6].

Complications of hemophilia A, particularly recurrent joint bleeding and hemophilic arthropathy, are among the most debilitating aspects of this condition. Hemophilic arthropathy, caused by repeated joint bleeding episodes, results in chronic pain and joint damage, significantly impacting patients’ quality of life. These challenges underscore the importance of effective management strategies to mitigate long-term complications (Table 1).

The severity of the disorder correlates with quality of life and treatment burden. Traditionally, individuals with hemophilia A were treated through factor replacement therapies, which, although effective, required frequent infusions and posed risks such as the development of inhibitors—antibodies which can neutralize the treatment. This underscores the need for innovative therapies to improve patient outcomes and quality of life. In recent decades, the management of hemophilia A has been revolutionized by advancements in gene therapy and innovative treatment modalities. Gene therapy aims to provide a long-lasting solution by introducing functional copies of the factor VIII gene into patients’ cells, potentially reducing or even eliminating the need for regular infusions. Clinical trials have shown promising results, with many patients achieving sustained factor levels and a significant reduction in bleeding episodes. Additionally, novel therapies, including bispecific antibodies and long-acting factor products, are reshaping treatment paradigms, offering patients more options and improved outcomes. These innovations not only enhance the quality of life for individuals affected by hemophilia A but also hold the promise of a future where the burden of this condition can be significantly alleviated.

The need for innovative therapies is further emphasized by the emotional and psychological challenges faced by patients. Coping with a chronic condition requires more than just medical intervention; it necessitates a holistic approach that includes emotional support, education, and community engagement.

This review highlights the remarkable progress in the understanding and treatment of hemophilia A, emphasizing the crucial role of gene therapy and innovative approaches in transforming the lives of those affected, while also acknowledging the historical journey that has brought us to this point. By focusing on the need for continued innovation, we can strive for a future where individuals with hemophilia A can lead healthier, more fulfilling lives.

## 2. Genetics of Hemophilia A

### 2.1. Overview and Clinical Manifestations of Hemophilia A

Hemophilia A (HA) is a monogenic disorder characterized by a lack or deficiency of factor VIII (FVIII), while, in hemophilia B (HB), there is a lack or deficiency of factor IX (FIX). Patients with mild hemophilia (level of FVIII between 6% and 40%) usually have no clinical signs and functional limitations; however, bleeding may occur in rare cases of trauma and surgery. As for the patients with moderate and severe hemophilia, with even lower plasma levels of FVIII, extensive hematomas, prolonged bleeding, and frequent joint and muscular bleeding appear [11].

### 2.2. Structure and Activation of FVIII

FVIII is a glycoprotein primarily produced by the sinusoidal endothelial cells of the liver, containing the following protein domains: A1, A2, B, A3, C1, and C2, along with acidic spacers. The heavy chain is represented by the A1-A2-B domain, while the light chain is formed by the A3-C1-C2 domain. The heavy and light chains remain bound to the von Willebrand factor (vWF) until FVIII is activated by thrombin, at which point the B domain, the acidic A3 region, and the von Willebrand factor dissociate from FVIIIa [12].

### 2.3. Genetic Basis and Mechanisms of Hemophilia A

The genes that code these factors are F8 and F9, located on the X chromosome [12]. In 1961, Mary Lyon used suggested that, in female mammals, one of the two X chromosomes is randomly inactivated during the early embryonic stage, ensuring that both males and females have only one active X chromosome. As both responsible genes are located on the X chromosome, they are subject to this inactivation pattern, initially called **lyonization** [13]. The inactivation of the X chromosome involves three well-characterized phases: initiation, spreading, and maintenance. The inactivation ratios can range from 50:50 distribution to 100:0 complete inactivation [14].

Males presenting this pathology are named **hemizygous**, and they have the full phenotype of the disease. In the meantime, the disease in females can result from multiple genetic causes [14,15]:

- **Homozygosity** (two identical hemophilia alleles—consanguinity, unrelated parental families, second mutation de novo, or two new mutations);
- **Compound heterozygosity** (two different hemophilia alleles inherited from each parent—unrelated parental families, second mutation de novo, or two new mutations);
- **Hemizygosity** (one hemophilia allele and no normal allele—Turner syndrome and mosaics, male karyotype, and X chromosome deletion);
- **Heterozygosity with skewed X chromosome** (a nonrandom inactivation in favor of the hemophilia allele) [16];
- **Genetic causes other than hemophilia** (the combined deficiency of factor V (Leiden) and factor VIII, von Willebrand disease type 2N, and combined vitamin K-dependent clotting factor);
- **Non-genetic causes** (acquired hemophilia due to inhibitors of FVIII or FIX, and vitamin K deficiency).

In about 30% of cases of both HA and HB, the condition is considered “sporadic”, where the newborn is the first in the family to be diagnosed; the mutation could arise in the child, mother, or maternal grandparents [15].

In the global literature, approximately 250 women and girls with severe or moderate disease have been reported, but the causes are insufficiently elucidated [14].

Both F8 and F9 genes are located on the long arm of the chromosome F8, closer to the end, at Xq28, and F9, more towards the centromere, at Xq27 [17]. The F8 gene is extremely large (∼180 kb) and structurally complex (26 exons). It also contains two nested genes, *F8A* and *F8B*, in the region of intron 22, responsible for the most frequent mutation in severe HA—intron 22 inversion (45% of cases of severe HA) [15,18]. A multicenter study conducted in the past 24 years [10] identified 36 distinct pathogenic/likely pathogenic variants among 4 apparently unrelated families—different types of defects occurred:

- Intron 22 inversion—39.1%;
- Intron 1 inversion—4.7%;
- Exon deletions, nonsense variants, frameshift variants, and splice site variants—31.3%;
- Missense and synonymous variants—25.0%.

In another study conducted by Yingdi Liu et al. (2023) [19], 131 pedigrees were included, out of which there were 35 intron 22-related gene rearrangements, 3 intron 1 inversions, 85 single-nucleotide variants and indels, 1 large insertion, and 7 large deletions.

Depending on the type of the genetic defect, the severity of the disease could be predicted. A significant disruption or absence of the FVIII protein is considered a **null variant** (e.g., the inversion of intron 22 and intron 1), present in most of the severe phenotypes, while for mild/moderate phenotypes, the highest prevalence of missense type is observed. Even though most of the genetic defects are irregularly distributed along the F8 gene, some hot spots have been identified—the intron 1, intron 2, and CpG dinucleotide hotspots [18]. However, 1–2% of severe HA cases are produced by deep intronic variation or *F8* disrupting genomic rearrangement, for which the sequencing of the whole F8 gene is needed [20].

Cases of HA with no mutations in their F8 gene have also been reported; therefore, other causes should be considered, such as the influence of microRNAs (miRNAs). MiR-374b-5p and miR-30c-5p, both expressed in the human liver alongside the FVIII protein, were analyzed in two patients with mild and moderate hemophilia A (HA) who showed no mutations in their F8 gene; it was proven that the overexpression of MiR-374b-5p and miR-30c-5p decreased FVIII expression, while an miR-30c inhibitor partially restored FVIII expression [18].

Since there is an X-linked inheritance of the disease, it is possible to identify obligate carriers. However, a negative family history is insufficient to exclude HA due to the possibility of sporadic mutations occurring or cases of maternal mosaicism [16].

## 3. Classical Treatment for Hemophilia

The aim of the classical treatment for hemophilia is to manage the acute bleeding episodes (episodic care) and prevent such episodes (prophylactic care). Gene therapy shows very promising results, but the classical approach for this condition is still chosen by most practitioners because of its efficiency over time, although it cannot meet all the requirements for calling hemophilia a curable disease.

The principle of the treatment protocols for hemophilia is to enrich the blood of the patient with the factor that they lack. This is called replacement therapy, which can be performed with standard half-life therapies, extended half-life therapies, plasma-derived therapies (fresh-frozen plasma, cryoprecipitate), and bypassing agents [21,22].

Besides replacement therapies, there are non-replacement therapies. Their target is not to directly supersede the clotting factor, but to bring other components that improve the functions of the blood.

### 3.1. Replacement Therapy

#### 3.1.1. Plasma-Derived Therapies

**Plasma-derived therapies** are represented by **fresh-frozen plasma** and **cryoprecipitate** (obtained from the centrifugation of the plasma), which were used mostly in the 1950s and 1960s, when recombinant DNA technology was not used to produce hemophilia treatments [23].

In 1958, Dr. Inge Marie Nilsson was the first to start the prophylaxis with **low-dose factor VIII infusions** to prevent eventual bleedings and joint damage. The results of this new approach were evaluated in a prospective randomized controlled trial, the Joint Outcome Study. The results showed that the prophylaxis with FVIII was efficient regarding the protection of joints and the prevention of cerebral hemorrhage and other types of bleeding. It was not a perfect procedure because joint damage could not be completely prevented, but, overall, it improved the condition in many children that had been treated with prophylactic FVIII infusions since childhood [24].

These treatments were possible because of the discovery of **cryoprecipitate** in the 1960s and, later, in the 1970s, **lyophilized clotting factor concentrates**. Despite their benefits, these concentrates were obtained from human plasma, which led to a historical disaster in the late 1970s and in the 1980s, when hemophilic patients were infected with HIV and hepatitis C virus [25].

Because of the short half-life of FVIII, there was a daily need for this type of treatment, which was given through vein puncture, affecting the patients’ quality of life [24]. Thus, there was a need for better and safer treatments, which led to new directions for hemophilia treatment in the 1990s—**the recombinant clotting factor concentrates**—and in the 2010s, **the longer half-life concentrates** [26].

Table 2 provides a concise summary of the key milestones in the evolution of hemophilia A treatment.

#### 3.1.2. Standard Half-Life Therapies

Standard half-life therapies (octocog alfa, lonoctocog alfa, simoctocog alfa, and moroctocog alfa) for type A hemophilia are recombinant factors and have an 8 to 12 h half-life, which translates, in clinical practice, in three or four infusions per week. The number of administrations of these infusions prevents the patients from becoming more adherent to the treatment [27,28].

#### 3.1.3. Extended Half-Life Therapies

Extended half-life therapies were developed through recombinant technology, such as immunoglobulin protein fusion, PEGylation, and fragment crystallization (Fc). They have 1.5–1.8 longer half-lives, which means fewer infusions per week and an increased compliance in patients [27].

Recombinant FVIII concentrates with extended half-life are used to treat **type A hemophilia.**

**Damoctocog alfa pegol** is an extended half-life recombinant FVIII obtained from conjugation with polyethylene glycol (PEGylated). It is site-specific and has been proven to be safe and efficient in the PROTECT VIII clinical trials. It has been approved in several countries for acute bleeding and prophylaxis for patients over 12 years of age [29].

**Efmoroctocog alfa** is an extended half-life recombinant FVIII obtained from protein fusion; it is approved for use and proven to be efficient and protective against bleeding episodes, with good tolerance from patients [30].

**Rurioctocog alfa pegol** is a third-generation PEGylated recombinant FVIII with extended half-life, used for prophylaxis, its low immunogenicity proven in studies. Another important aspect is the improvement of joint function by increasing collagen turnover and remodeling [31,32].

**Turoctocog alfa pegol** is also a PEGylated recombinant FVIII with extended half-life, proven to be efficient in clinical trials. Like rurioctocog alfa pegol, it was tested on patients that had been previously untreated for hemophilia [33,34].

**Efanesoctocog alfa** is a recent treatment that was regulatorily approved in the USA and Japan in 2023, and it is a combination between FVIII and the von Willebrand factor, which elongates its half-time by 3–4 times. It is efficient, and its weekly administration makes it very comfortable for patients [35,36].

### 3.2. Non-Replacement Therapy

#### 3.2.1. Bispecific Monoclonal Antibody

**Emicizumab** is a bispecific monoclonal antibody that binds to factor IX and factor X, mimicking, in this way, FVIII and replacing its function [22].

**Mim8** is a next-generation bispecific monoclonal antibody that also binds to F IX and F X and generates a higher level of thrombin than emicizumab, currently in phase ½ studies [37].

#### 3.2.2. Other Therapies for Specific Settings

**Desmopressin** is the synthetic form of vasopressin and helps in mild hemophilia. **Aminocaproic acid** and **tranexamic acid** (lysine analogs) are antifibrinolytic drugs that help prolong blood clotting during dental surgeries. **Fibrin glue** is also used for dental procedures. **Prothrombin complex concentrates** contain, in addition to prothrombin, other clotting factors (II, VII, IX, and X) [21,22].

**Fitusiran** is a small interfering RNA, administered subcutaneously and currently in phase 3 clinical trials, that shows promising results for male patients ≥ 12 years of age, lowering their antithrombin levels and allowing procoagulat factors to increase. It is used for the prophylaxis of hemophilia A or B with/without inhibitors, with 80 mg once a month, for 7 months [38].

**Concizumab** is an Ig4 monoclonal antibody that binds to the K2 domain of the tissue factor pathway inhibitor (TFPI), which prevents the activation of F IX. Therefore, concizumab stops the action of TFPI and leads to the enhancement of thrombin levels. **Marstacimab** and **Befovacimab** have a similar mechanism of action to concizumab, and all of them are currently in clinical trials [37].

**SerpinPC** is an inhibitor of the activated protein C, which degrades the activated F V and F VIII. This new treatment, currently in phase 1/2 clinical trials with volunteers, is an improved version of serpins (serine protease inhibitors) that naturally inhibit activated protein C and promote thrombin levels [37].

### 3.3. Acquired Hemophilia

Acquired hemophilia is a rare, autoimmune disease in which FVIII is attacked by the patient’s autoantibodies. There are some treatment options, comprising the following:

- Human recombinant FVII (for patients over twenty years of age);
- Recombinant porcine FVIII;
- Immunosuppressive therapy;
- Emicizumab, used in some studies, which is a monoclonal antibody designed to mimic the function of FVIII, as a second-line treatment for patients with acquired hemophilia. However, selecting the appropriate patients is very important because of the associated risk of thrombosis and thromboembolism. [39,40,41].

### 3.4. Bypassing Agents

Bypassing agents are clotting factors used in patients with hemophilia who have developed inhibitors. These agents help bypass the need for the deficient clotting factor, which is ineffective because the patient has developed antibodies against it [22].

**Hemophilia with inhibitors** represents a complication of the condition, characterized by the development of antibodies against factor replacement therapy. In this case, the body produces antibodies that interfere with the effectiveness of replacement therapy. These inhibitors typically develop in the patient’s blood after the start of the treatment. The treatments for this complication are the following:

- Human recombinant activated coagulation FVII (**eptacog beta**—new recombinant activated human FVII; and **marzeptacog alfa**—subcutaneous activated recombinant human FVII);
- Human recombinant FVIII (**simoctocog alfa**) [40,41,42,43,44];
- Emicizumab.

## 4. Gene Therapy for Hemophilia

### 4.1. Gene Therapy Advances in Hemophilia

As a monogenic disease, with easily measurable laboratory and clinical endpoints, hemophilia has become one of the main targets for gene therapy, with the first study being conducted for HB [45].

**Gene therapy** has been taken into consideration as a possible treatment for HA for the past two decades; consequently, numerous gene therapy strategies have been exhaustively researched. The process of cloning F8 and F9 genes was first studied in 1980; the extensive research conducted over the past 30 years has led to the development of certain approved therapies, based on the efficacy and safety data collected. Roctavian® (valoctocogene roxaparvovec), an adeno-associated viral AAV5 vector carrying B domain-deleted FVIII complementary DNA, has been approved by the European Commission and the FDA for the treatment of severe hemophilia A in adult patients [46].

### 4.2. Efficacy and Safety of AAV-Based Gene Therapy

Currently, the most used therapeutic approach consists of single infusions of a viral vector, **a recombinant adeno-associated virus (AAV) vector**, which result in the transduction of a gene coding for the deficient factor into patient hepatocytes. The virus consists of a capsid that surrounds a DNA sequence or a transgene. Being unable to replicate without a helper virus, the insertion of AAV is safe and non-pathogenic [47]. The F8 gene needs to be truncated to be small enough to be inserted into the AAV vector; usually, a sequence encoding a hemostatically non-functional domain is removed [45]. Single-stranded AAV (ssAAV) vectors deliver a single-stranded DNA sequence into the nucleus, where the host cell’s machinery synthesizes a complementary strand, leading to the formation of the functional gene [47].

Particularly important in the process of decision making when choosing gene therapy as a treatment is the fact that AAV-mediated gene therapy is a one-time irreversible process, and hepatic adverse reactions occur after its administration [48]. Raised ALT (alanine aminotransferase) and AST (aspartate aminotransferase) have been reported as the most common side effects [49]. A study regarding the effects on dog liver 10 years after the therapy showed little modifications in the liver biopsy: the overall architecture was normal, with no evidence of malignancy, but with centrilobular/midzonal hepatocellular hypertrophy [45]. This condition is ameliorated with corticosteroids, whose administration should be carefully monitored. Moreover, anticoagulants seem to also be required, as thrombotic events have been recently reported as a side effect of gene therapy with AAV [48].

### 4.3. Valoctocogene Roxaparvovec

As previously mentioned, **Valoctocogene roxaparvovec (AAV5-hFVIII-SQ)** is a currently approved gene therapy product; therefore, it has been extensively studied. BMN70 (NCT02576795) was the first study to test the efficacy of the AAV5-co-BDD-FVIII vector in nine patients. In the high-dose group (n = 7, 6 × 10^13^ vg/kg), the FVIII levels increased to over 5 IU/dL between weeks 2 and 9, reaching normal levels (FVIII > 50 IU/dL) in six out of seven patients after week 20. FVIII expression remained stable after 1 year (median FVIII 77 IU/dL, range 19–164 IU/dL). The ALT levels were elevated in eight out of nine participants, with no immune response and adequate corticosteroid administration [50].

The largest study of Valoctocogene roxaparvovec (AAV5-hFVIII-SQ) was an open-label, single-group, multicenter phase 3 trial involving 134 adult patients. These patients had no pre-existing AAV antibodies and received an infusion of the treatment. Among the 132 HIV-negative participants, the mean factor VIII activity increased by 41.9 IU/dL at weeks 49–52 (*p* < 0.001), with a median change of 22.9 IU/dL. In 112 participants from a non-interventional study, factor VIII use and treated bleeding decreased by 98.6% and 83.8%, respectively (*p* < 0.001). All participants had at least one adverse event, such as headache (38.1%) and nausea (37.3%), and 16.4% experienced serious events. Elevated alanine aminotransferase levels occurred in 85.8% and were managed with immunosuppressants [51].

Valoctocogene roxaparvovec was also compared with other therapeutic alternatives: compared to emicizumab, the annual bleeding rate was statistically significantly lower with valoctocogene roxaparvovec (RR = 0.55, 95% CI [0.33–0.93]), and higher proportions of participants had no treated joint bleeds (OR = 2.75, 95% CI [1.20–6.31]) and no treated bleeds (OR = 3.25, 95% CI [1.53–6.90]) [52].

### 4.4. Other Products Used for AAV-Gene Based Therapy

Additional AAVs have been developed in the past decade. In the ongoing phase 1/2, dose-ranging Alta study, four sequential cohorts of male participants with severe hemophilia A received a single IV dose of **giroctocogene fitelparvovec**, a recombinant AAV serotype 6 vector. Apart from the increase in ALT and AST, hypotension and pyrexia were reported in one participant within 6 h of infusion and were resolved within 24 h of infusion. At the highest dose level (3 × 10^13^ vg/kg; n = 5), the mean FVIII activity was 42.6% at week 52 and 25.4% at week 104, with ranges of 7.8–122.3% and 0.9–71.6%, respectively, based on a chromogenic assay [53].

**Dirloctocogene samoparvovec** is another vector studied. In the phase 1/2 trial, the FVIII activity levels, as measured by a one-stage assay (OSA), ranged from 4 to 22 IU/dL for 16 out of the 18 participants [54]. Another phase 1/2 study involving BAY 2599023 (AAVhu37.hFVIIIco-SQ, DTX 201) enrolled nine patients across three cohorts (0.5 × 10^13^ genetic copies [GC]/kg; 1 × 10^13^ GC/kg; and 2 × 10^13^ GC/kg), observing sustained FVIII expression for over 23 months [55].

Sangamo Therapeutics also released data from the SB-525 study (NCT03061201) using an AAV6-BDD-FVIII vector, showing dose-dependent FVIII elevation, in April 2019. In the 3 × 10^13^ vg/kg group, two patients achieved normal FVIII levels (140% and 94%) by week 6. The vector is now extensively researched in other studies [45].

### 4.5. Limitations of AAV-Based Gene Therapy

As expected, all the aforementioned benefits came along with some limitations [47]. The most impactful one is the pre-existence of **anti-capsid neutralizing antibodies**, which would make the treatment inefficient. Treatment success in these patients is currently undetermined since they were excluded from previous trials. In addition, **the turnover of the hepatocyte is also affected**, resulting in decreased transgene-induced factor production over time and the need for AAV re-induction. Finally, increased production of the coagulation factors is considered to imply **higher cellular stress and toxicity**.

A major challenge is derived from the fact that the gene therapy vectors may increase the risk of cancer development; further studies are needed to exclude this [56].

### 4.6. Other Therapies—HIV-Derived Lentiviral Vectors and CRISPR-Cas9 Therapy

Taking these limitations into account, other strategies were tested. For example, HIV-derived lentiviral vectors (LVs) may represent a feasible alternative to AAV due to the efficient integration of LVs in the host cells’ genome and the larger packaging capacity that may allow LVs to transfer the whole genetic material. However, further testing is required [57].

In terms of safety, nonviral vectors are superior to viral ones in the process of delivering genome editing tools. The transient expression of CRISPR-Cas9 is sufficient to edit the targeted genes, presenting less adverse immune effects. CRISPR—Cas9 has also been tested for the correction of genetic defects, such as F8 intron 1 inversion. A donor plasmid including the human alpha 1-antitrypsin (hAAT) promoter, exon one, and the splicing donor site (SD) based on the homology-mediated end joining (HMEJ) strategy was used, targeting hemophilia A patient-derived induced pluripotent stem cells (HA-iPSCs), which eventually differentiated into hepatocyte-like cells (HPLCs). The supernatant from HA-iHPLCs, T-26-iHPLCs, T-73-iHPLCs, and N-iHPLCs was collected for FVIII antigen detection using ELISA. Higher level of FVIII were detected in T-26-iHPLCs than HA-iHPLCs; immunofluorescence confirmed FVIII expression in T-26-iHPLCs, but not in HA-iHPLCs, indicating the successful restoration of FVIII expression in gene-corrected iPSC-derived HPLCs [58].

## 5. Global Concerns and Policies

Treatment options for hemophilia patients are expanding, with promising results expected in the near future. Access to treatment should be optimized in terms of locations, logistics, and public funding, and this is often made possible by the collaboration between governmental institutions and non-governmental organizations representing the patients’ best interest.

### 5.1. Global Approach

Although Europe and the USA have strong regulations regarding treatment types and the medical institutions which can provide them, there is a considerable difference between countries with a high level of development and those with low incomes, with a lag of more than 40 years. The rate of diagnosis varies between 100% in high-income countries to 12% in low-income countries. These data show a dramatic disparity between nations around the world, given the fact the World Bleeding Disorders Registry of the World Federation of Hemophilia collected these data from 87 centers all over the world, with 67% out of 10,276 patients being from low–middle-income countries [59].

The World Federation of Hemophilia elaborates guidelines for the management of hemophilia, which are revised by the World Health Organization (WHO) in the annual Review Process of the WHO Model List of Essential Medicines (EML) and the Model List of Essential Medicines for Children (EMLc), where the WHO gives directives for the indications, limitations, dosage, types, and alternatives of medicines, as well as sources of information. In this manner, there is communication between the highest authority in the medical field and the most important organization for hemophilia, which is closer to patients’ needs and requirements [60].

### 5.2. Treatment Approach in the United States of America

In the USA, the **National Hemophilia Program**, according to the Health Resources and Services Administration, consists of eight **Regional Hemophilia Networks (RHNs)** that supervise a regional network of **Hemophilia Treatment Centers (HTCs)** across the United States. The RHNs and the HTCs receive support from the **National Hemophilia Program Coordinating Center (NHPCC)**. These centers are very important for patients with hemophilia because of their expertise and their focus on research and complete care. In the USA, there are over 40,000 patients with hemophilia, and inadequate care can lead to higher costs due to the frequency of hospitalizations and the absence of these patients from the work field; with proper care, they could lead more active lives and proceed along their desired careers [61].

### 5.3. Treatment Approach in Europe

In Europe, there are regulations regarding centers for hemophilia care; these are in the European guidelines for the certification of **hemophilia centers** by the **European Hemophilia Network (EUHAD)**, in collaboration with the **European Association for Hemophilia and Allied Disorders (EAHAD)** and the **European Hemophilia Consortium (EHC)**. These are basic standards of practice that can be upgraded according to the specific governmental laws and regulations of each European country. The aim is to introduce this practice in various countries on this continent to a satisfactory standard and give equal chances to patients all over Europe.

Similarly to the USA, Europe registers its patients and provides them with medical care in regional and centralized institutions. European hemophilia centers are divided in two major categories, **European hemophilia treatment centers (EHTCs)** and **European hemophilia comprehensive care centers (EHCCCs)**.

EHTCs are local, should provide care to at least 10 patients, and should be able to diagnose, treat, follow-up on, and rehabilitate them. These centers should manage patients during emergencies and regular check-ups. EHTCs can access other medical services that are meant to improve patients’ overall quality of life (physiotherapy, dental care, clinical psychology, social services, and genetic counseling) or manage other multidisciplinary needs such as hepatology, infectious diseases, surgeries, etc.

EHCCs are tertiary referral centers that provide services at the highest level, like the EHTCs which they supervise, and should be able to have at least 40 patients registered. These centers are research-oriented and could be involved in clinical trials [62].

Europe also has a pharmacovigilance program called the **European Hemophilia Safety Surveillance (EUHASS)** [63].

**Euro Blood Net** is the **European Reference Network on Rare Hematological Diseases**, which is part of the European Reference Networks created by a **European Parliament** directive for conditions which require highly specialized medical care. There are centers enrolled in the Euro Blood Net in many European nations, such as Belgium, France, Germany, Italy, Portugal, Spain, The Netherlands, the United Kingdom, and Bulgaria. Unfortunately, Romania does not have any reference center, expert, or member institutions, although it has adequate regulations around hemophilia care [64].

Table 3 provides an overview of the main categories of treatment for hemophilia A.

### 5.4. Treatment Approach in Romania

In Romania, the National Program for the Treatment of Hemophilia and Thalassemia aims to provide essential care and support for individuals affected by these inherited blood disorders [65]. The inclusion criteria for this program are regulated by a ministerial order from the Ministry of Health and the President of the National Health Insurance House of Romania (C.N.A.S.), establishing the legal framework, guidelines, and standards of care which must be followed in the diagnosis, treatment, and monitoring of patients with hemophilia and thalassemia. The ministerial order ensures the allocation of the necessary resources for treatment and the organization of specialized centers nationwide.

The program includes various hospital units:

- **For the non-surgical treatment of patients,** if they meet the specific evaluation criteria;
- **For the surgical treatment of patients**, including the Fundeni Clinical Institute, the Timișoara County Emergency Clinical Hospital, the “Louis Ţurcanu” Emergency Clinical Hospital for Children Timișoara, the Craiova County Clinical Hospital, the Târgu Mureș County Emergency Clinical Hospital, the “Prof. Dr. I. Chiricuță” Oncology Institute Cluj-Napoca, the “Sf. Spiridon” County Emergency Clinical Hospital Iași, the “Sfânta Maria” Emergency Clinical Hospital for Children Iași, the “Dr. Carol Davila” Central Military Emergency University Hospital Bucharest, and the Timișoara Municipal Emergency Clinical Hospital;
- Other health facilities belonging to ministries, with their own healthcare network.

In April 2022, 1230, patients with congenital or acquired hemophilia were enrolled in the national program. The distribution and the therapeutic approach applied are shown below (Table 4): 

All the aforementioned treatments were reimbursed through the program, making them highly accessible to the entire patient population. Currently, gene therapy is not available in Romania.

## 6. Short Comments

The evolving landscape of hemophilia A treatment highlights the substantial strides made in understanding and managing this complex disorder. From the early recognition of hemophilia’s hereditary pattern to the introduction of gene therapy, scientific advances have provided hope for improved, long-term outcomes, with reduced burdens and risks. When deciding which treatment to apply, the patient should be directly involved in making this decision, since multiple choices are available and a balance between classical treatment approaches and emerging therapies should be kept.

There are certainly challenges in detecting and treating both genetic and acquired forms of hemophilia; novel treatment modalities—including gene therapy, extended half-life factor products, and non-factor replacement therapies like emicizumab—are redefining patient care. From now on, research should be oriented towards improving patients’ quality of life, and larger human trials should be conducted to test novel gene therapies while respecting ethical principles. However, the restriction to articles published or translated solely in English represents a limitation of our review, as it may have excluded valuable insights published in other languages.

As hemophilia is advancing globally, questions regarding patients’ access to optimal care start to arise. There is an inconsistency, especially between high-income countries, where diagnosis rates are nearly universal, and low-income countries, where access to treatment lags significantly behind. Efforts by organizations like the World Federation of Hemophilia and the WHO aim to standardize care through guidelines and essential medicine lists, though disparities persist. In both Europe and the USA, regulatory frameworks and different stakeholders, such as the National Hemophilia Program in the U.S. and European hemophilia centers, are designed to deliver the best standardized care. Romania serves as an example of how a well-organised national funding programme can support various treatments for this disorder. However, the lack of gene therapy options and the absence of a Euro Blood Net reference centre highlight areas for improvement. Collaborative efforts between government and non-government organisations are needed to address these gaps.

## 7. Conclusions

Significant advancements have been achieved in hemophilia management over the past few years. The significant shift in treatment, from traditional factor replacement to innovative gene therapies, offers patients new treating solutions, but some of them need to be further investigated. As scientists’ interest is currently oriented towards developing more individualized treatments, various stakeholders are tackling a new challenge: ensuring equitable access to these advancements.

## Figures and Tables

**Table 1:** Overview of clinical presentation, pathophysiological characteristics, common complications, and treatment approach in hemophilia A [1,2,8,9,10].

Severity Level	Clotting Factor Levels (%)	Age of Onset	Typical Clinic Presentation	Pathophysiological Characteristics	Common Complications	Treatment Approach
MILD	6–40% of normal level	Late childhood/ adulthood (post trauma or surgery)	Prolonged bleeding after trauma/ surgery Frequency of bleeding: uncommon	Partial preservation of factor VIII activity; adequate thrombin generation in most situations	Rare joint damage; minimal bleeding risks	Factor VIII replacement therapy when needed (during trauma or surgery)
MODERATE	1–5% of normal level	Childhood	Bleeding after minor injuries; occasional hemarthrosis Frequency of bleeding: 4 to 6 times/y	Insufficient factor VIII activity for adequate thrombin generation after mild trauma	Chronic joint disease; rare intracranial hemorrhage	Prophylactic or on-demand therapy
SEVERE	<1% of normal level	Infancy (spontaneous bleeding episodes)	Spontaneous bleeding in joints, muscles, or brain Frequency of bleeding: 2 to 4 times/mo	Near absence of factor VIII activity; inability to form stable fibrin clots	Hemarthroses, ecchymoses, intracranial hemorrhages, joint damage, and prolonged bleeding after injury	Routine prophylaxis with clotting factor
NORMAL	50% to 150% of normal activity, or 0.5–1.5 IU/mL These levels may vary slightly depending on the laboratory and method of measurement.	N/A	No abnormal bleeding episodes	Fully functional clotting factor VIII (normal factor VIII levels, adequate coagulation)	None	No treatment needed

**Table 2:** Summary of the main events in the evolution of treatment for hemophilia A.

Decade/Year	Treatment
1950s	Fresh-frozen plasma
1958	Low-dose factor VIII infusions
1960s	Cryoprecipitate
1970s	Lyophilized clotting factor concentrates
1990s	Recombinant clotting factor concentrates
2010s	Extended half-life concentrates

**Table 3:** Summary of the main categories of treatment for hemophilia A.

Replacement/Non-Replacement	Type of Treatment	Name of Treatment
Replacement therapy	Plasma-derived therapies	Fresh-frozen plasma Cryoprecipitate Low-dose factor VIII infusions Lyophilized clotting factor concentrates
	Standard half-life therapies	Octocog alfa Lonoctocog alfa Simoctocog alfa Moroctocog alfa
	Extended half-life therapies	Damoctocog alfa pegol Efmoroctocog alfa Rurioctocog alfa pegol Turoctocog alfa pegol Efanesoctocog alfa
	Bypassing agents	
Non-replacement therapy	Bispecific monoclonal antibody	Emicizumab
	Others	Desmopressin Aminocaproic acid Tranexamic acid Fibrin glue Prothrombin complex concentrates

**Table 4:** Distribution of patients with hemophilia in Romania in April 2022 and the treatments they received (the data were collected from the latest report of the National Program for the Treatment of Hemophilia and Thalassemia in Romania).

Patients	Treatment
congenital hemophilia without inhibitors/von Willebrand disease	continuous prophylactic substitution (n = 211) intermittent prophylactic substitution (n = 325) “on-demand” treatment (n = 543)
congenital hemophilia with inhibitors	Secondary prophylaxis in the long term (n = 19) secondary prophylaxis in the intermediate/short term (n = 18) bleeding cessation treatment (n = 55)
congenital hemophilia with or without inhibitors/von Willebrand disease, referred for surgeries	substitution treatment for surgical/orthopedic procedures (n = 50)
acquired symptomatic hemophilia	substitution treatment (n = 9)

## Data Availability

Not applicable.
